# P-2063. Safety and Immunogenicity of Booster Vaccination Against COVID-19 with Whole-SARS-CoV-2-Virion Inactivated Vaccine KD-414: A Phase 1 Trial in Japan

**DOI:** 10.1093/ofid/ofae631.2219

**Published:** 2025-01-29

**Authors:** Michiko Koga, Masahiro Nojima, Kengo Sonoda, Ken Ishii, Yoshihiro Kawaoka, Fumitaka Nagamura, Hiroshi Yotsuyanagi

**Affiliations:** The University of Tokyo, Minato-ku, Tokyo, Japan; The University of Tokyo, Minato-ku, Tokyo, Japan; KM Biologics Co., Ltd., Kikuchi-shi, Kumamoto, Japan; The University of Tokyo, Minato-ku, Tokyo, Japan; University of Wisconsin, Madison, WI; The University of Tokyo, Minato-ku, Tokyo, Japan; The University of Tokyo, Minato-ku, Tokyo, Japan

## Abstract

**Background:**

SARS-CoV-2 mRNA vaccines induce effective immunity, but wane over time. With COVID-19 now endemic, there is a growing population with hybrid immunity. Booster immunization with a vaccine that offers fewer adverse events and sustained immunity is imperative.

**Figure:**
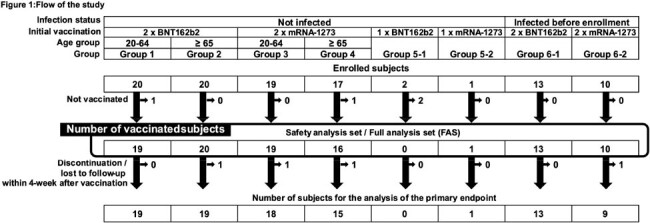

**Methods:**

We conducted an open-label, dose-fixed, multi-group, single-center study for adults (≥20 years old) in Japan, administering a single booster dose of KD-414, a whole-SARS-CoV-2-virion inactivated vaccine, after primary vaccination with mRNA vaccines (BNT162b2, mRNA-1273), including post-SARS-CoV-2 infection (jRCT1031210517). Safety and immunogenicity were assessed using the geometric mean titer (GMT) of serum neutralizing antibodies (NA) against the vaccine strain [Wuhan strain (W)], and an Omicron strain (BA1). T-cell function in peripheral blood mononuclear cells (PBMCs) was also evaluated after stimulation with SARS-CoV-2-S, M, and N peptide vaccine strains using a multiplex system. Subjects were observed for 52 weeks if they remained uninfected with SARS-CoV-2 and did not receive additional vaccinations.

**Figure:**
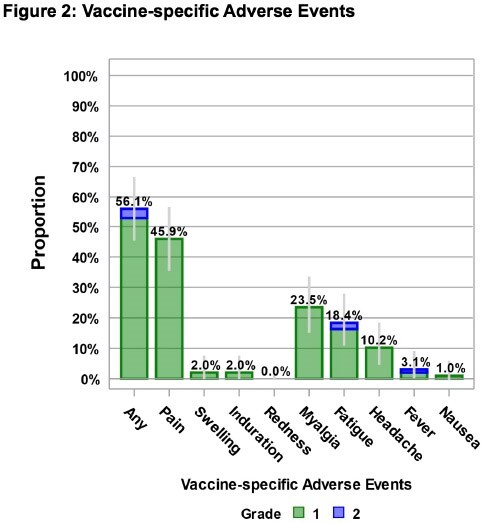

**Results:**

Among the 102 adult volunteers enrolled (Figure 1), KD-414 was well tolerated, with vaccine-specific adverse events predominantly grade 1 (Figure 2). The GMT of W strain NA before booster injection varied, resulting in categorization into low, medium, and high groups based on NA. The proportion of subjects with at least a 4-fold increase in NA in the low group increased to 54.5% (18/33) at 4 weeks (Figure 3). IFN-γrelease by PBMCs stimulated with each peptide correlated with W strain NA (Figure 3). The proportion of subjects with NA upregulation of 10 or more in the medium and high groups remained at 100% until 52 weeks, whereas the low group showed a decrease to 92.3% and 66.7% at 26 weeks and 52 weeks, respectively (Figure 4). Regarding the GMT of BA1 strain NA, previously infected subjects exhibited higher levels (data not shown). The proportion of subjects with NA upregulation of 10 or more in the medium and high groups also remained at 100% until 52 weeks (Figure 4).

**Figure:**
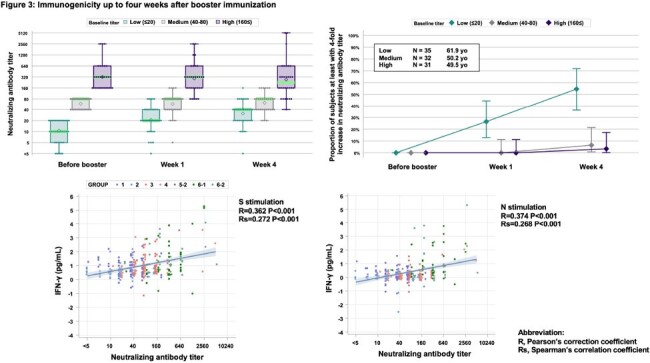

**Conclusion:**

A single booster dose of KD-414 showed good tolerability and immunogenicity, suggesting its potential as s safe and novel SARS-CoV-2 booster vaccine for individuals with hybrid immunity.

**Figure:**
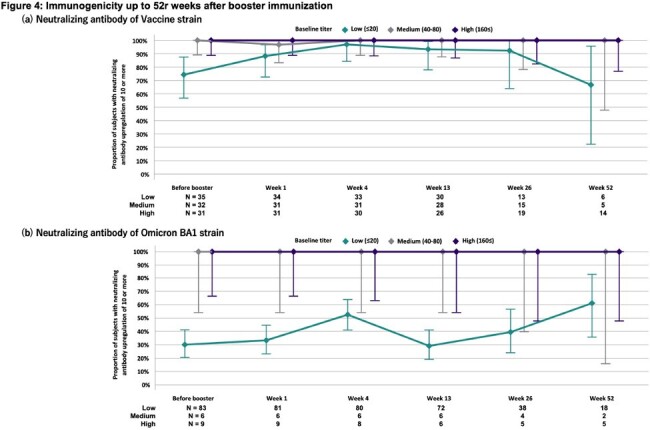

**Disclosures:**

Michiko Koga, MD,PhD, KM biologics: Grant/Research Support Masahiro Nojima, M.D.,Ph.D., KM biologics: Grant/Research Support Kengo Sonoda, PhD, KM Biologics Co., Ltd.: Kengo Sonoda is an employee of KM Biologics Co., Ltd. Ken Ishii, M.D., Ph.D., KM biologics: Grant/Research Support Yoshihiro Kawaoka, DVM, Ph.D., Daiichi Sankyo: Grant/Research Support|FluGen: Stocks/Bonds (Private Company)|KM Biologics: Grant/Research Support Fumitaka Nagamura, M.D., Ph.D, DeNA Life Science: IRB member Hiroshi Yotsuyanagi, MD PhD, Japanese Society of Infectious Disease: President|Shionogi & Co., Ltd.: Board Member|Shionogi & Co., Ltd.: Lecture fees, travel and meeting support, and Chairs in sponsored symposiums|ViiV Healthcare: Lecture fees; Chairs in sponsored symposiums

